# Post COVID-19 among young adults– prevalence and associations with general health, stress, and lifestyle factors

**DOI:** 10.1186/s12889-025-22522-9

**Published:** 2025-04-09

**Authors:** Sandra Ekström, Ida Mogensen, Maria Ödling, Antonios Georgelis, Anne-Sophie Merritt, Sophia Björkander, Erik Melén, Anna Bergström, Inger Kull

**Affiliations:** 1 Center for Occupational and Environmental Medicine, Region Stockholm, Stockholm, 113 65 Sweden; 2 Department of Clinical Science and Education, Karolinska Institutet, Södersjukhuset, Stockholm, 118 83 Sweden; 3 Institute of Environmental Medicine, Karolinska Institutet, BOX 210, 171 77, Stockholm, Sweden; 4 Department of Women’s and Children’s Health, Karolinska Institutet, Stockholm, 171 77 Sweden; 5 Sachs’ Children’s and Youth Hospital, Södersjukhuset, Stockholm, 118 61 Sweden

**Keywords:** General health, Lifestyle factors, Post COVID-19, Stress, Young adults

## Abstract

**Background:**

The prevalence of post COVID-19 condition (PCC) after mild infection among young adults is largely unknown, as are its impact on health and lifestyle factors.

**Objective:**

To assess the prevalence of PCC among young adults and its impact on general health, stress, and changes in lifestyle factors three years after the onset of the pandemic.

**Methods:**

The study population (*n* = 2,098) included participants from the population-based cohort BAMSE (aged 27–30 years). PCC symptoms and changes in lifestyle factors during the pandemic were assessed in a questionnaire distributed in September–December 2023 and analyzed cross-sectionally. Stress, physical activity, and general health were also assessed pre-pandemic (2016–2019) and analyzed longitudinally. PCC was defined as ≥ 1 symptom lasting for ≥ 2 months after COVID-19.

**Results:**

In total, 1,577 (75.5%) reported previous COVID-19. Among these, 166 (10.5%) reported previous and 62 (3.9%) ongoing PCC. The most common ongoing symptoms were altered smell/taste, psychological symptoms, and fatigue. Both pre- and post-pandemic general health differed significantly in relation to PCC in cross-sectional analyzes (all *p* < 0.05), with the lowest health reported by those with ongoing PCC. Participants with ongoing PCC also had a reduction in well-being in longitudinal analyses (*p* = 0.04). This group also reported more adverse changes in lifestyle factors and health during the pandemic such as reduced physical activity (*p* < 0.001) and worsened dietary habits (*p* = 0.03). However, there was no significant difference in the longitudinally measured perceived stress scale among individuals with PCC.

**Conclusions:**

Almost 4% of young adults with previous self-reported COVID-19 had ongoing symptoms of PCC three years after the onset of the pandemic. This group reported poorer health and more adverse changes in lifestyle factors than participants without PCC. Targeted healthcare interventions for young adults with PCC are warranted.

**Supplementary Information:**

The online version contains supplementary material available at 10.1186/s12889-025-22522-9.

## Background

Although the prevalence of severe, acute corona virus disease-19 (COVID-19) has decreased since the beginning of the pandemic in 2020, it was still ranked as the 10th leading cause of death in the US in the year 2023 [1–4]. In addition to acute effects, the infection can also lead to long-term sequelae, so called “post COVID-19 condition (PCC)” or “long covid” [5–8]. The World Health Organization has proposed the following definition of PCC: “The continuation or development of new symptoms 3 months after the initial SARS-CoV-2 [severe acute respiratory syndrome coronavirus 2] infection, with these symptoms lasting for at least 2 months with no other explanation” [9].

In a recently published meta-analysis, the pooled prevalence of PCC worldwide was estimated at 42% [10]. In non-hospitalized patients followed for up to 40 weeks after infection, the prevalence has been reported to be somewhat lower, ranging between 7.5% and 41% [11]. In contrast, one study on non-hospitalized adolescents found that PCC symptoms were not more common compared with among controls without PCC at six months after infection [12]. How prevalent PCC symptoms are beyond that time frame is less studied.

PCC has been found to be more common among females than males [13–17]. Moreover, several chronic diseases, including cardiovascular disease, obesity and psychiatric disease have been identified as risk factors for PCC [7, 11, 13, 18, 19]. In addition, asthma has been linked to both more prolonged respiratory symptoms after SARS-CoV-2 infection and health-related anxiety in connection with COVID-19 [20, 21].

After the pandemic, concerns have been raised regarding how COVID-19 and PCC affect general health and lifestyle. Studies have found more severe COVID-19 to reduce the ability to perform activities of daily living [22], and reduced health-related quality of life and physical function up to half a year after COVID-19 [23, 24]. However, most studies have focused on middle-aged or older patients and mainly evaluated physical function rather than general health and lifestyle habits in daily life [23].

If, and how, PCC influences health and lifestyle in younger individuals is less known. COVID-19 generally causes milder disease in young adults [25]. Despite this, PCC symptoms have been reported to be common in this age group too [20]. Moreover, in a young population, lifestyle habits can have a large impact on future health, making studies on factors influencing lifestyle and health in young adulthood important.

In this study, the aims were to assess the prevalence of previous and ongoing PCC symptoms among young adults from a population-based cohort, three years after the onset of the pandemic, and to investigate the impact of PCC on general health and changes in lifestyle factors (e.g. physical activity and diet) after the pandemic.

## Methods

### Study design and study population

The study population originates from the population-based birth cohort BAMSE. The participants were recruited in 1994–1996, when all children born in four pre-defined municipalities in the northwestern and central parts of Stockholm, Sweden were identified through the population register and invited to participate (*n* = 7,221). Of these, 477 could never be reached and 1256 were excluded based on to pre-defined exclusion criteria: the family planned to move within one year of the study start, the parents had insufficient knowledge of the Swedish language, the family had a seriously ill child, or a sibling was already recruited into the study. In total, 5488 children were eligible. Of these 4,089 (75%) agreed to participate and were included in the cohort.

The participants have been followed through repeated questionnaires and clinical examinations. The present study is based on data from a follow-up at participant age 22–24 years (conducted in 2016–2019, i.e., “pre-pandemic”), referred to as the “24-year follow-up” [26] and part four of a COVID-19 focused follow-up (participant age 27–30, conducted in 2023, i.e., “post-pandemic”), referred to as the “COVID-19 phase 4 follow-up” [20, 27].

The 24-year follow-up included a questionnaire (*n* = 3,064, 75% response rate) focusing on asthma and allergic symptoms, lifestyle,- and socioeconomic factors, as well as a clinical examination (*n* = 2,250). The COVID-19 phase 4 follow-up invited participants who completed the 24-year questionnaire and had provided a valid e-mail address (*n* = 2,981). This follow-up included a questionnaire (*n* = 2,101, 71% response rate, 59.3% females) focusing on COVID-19, PCC symptoms, general health, and lifestyle factors. The current study population includes all participants who provided information on COVID-19 and PCC symptoms (*n* = 2,098), Supplement Fig. 1.

**Fig. 1:**
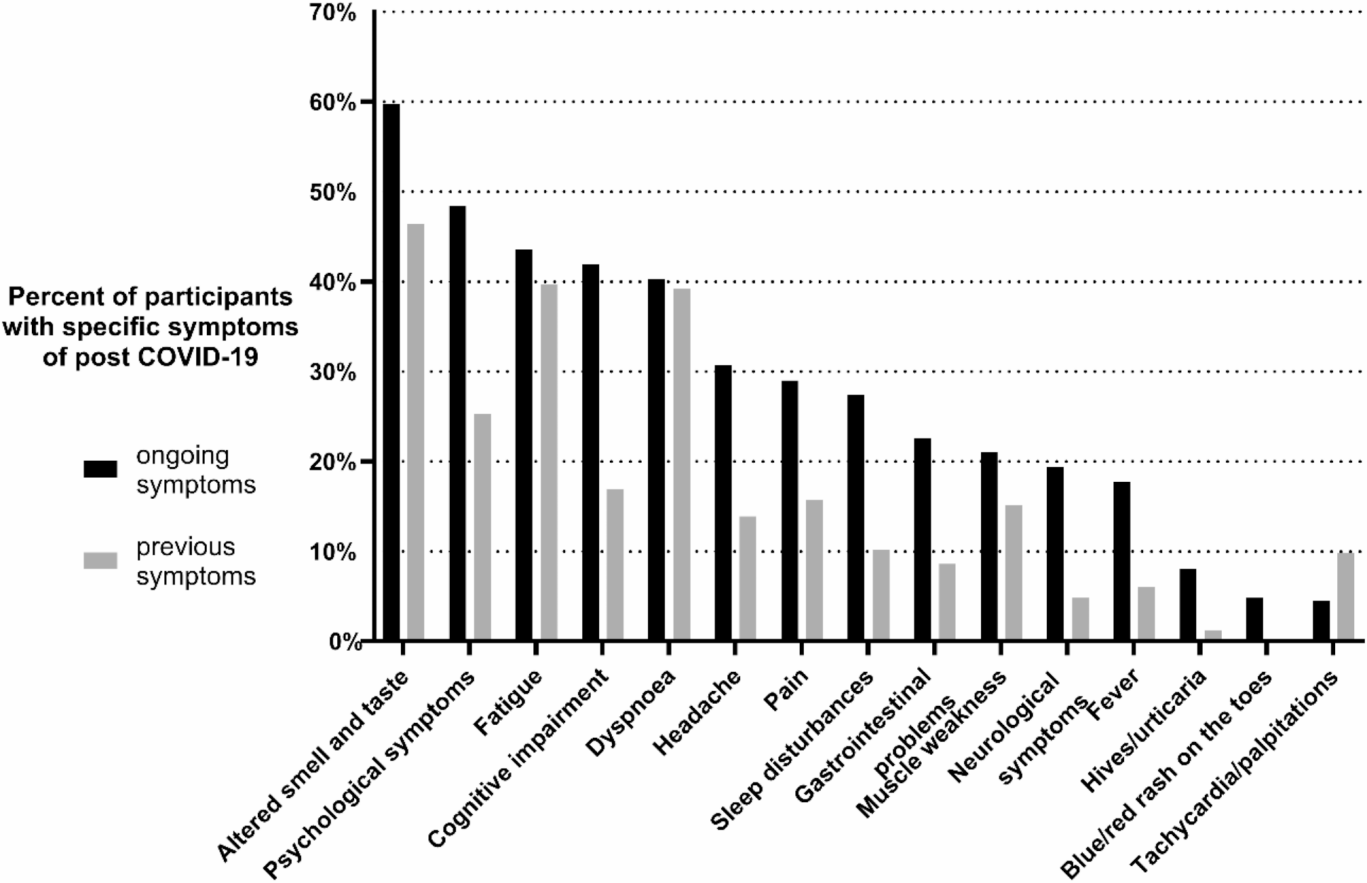
Prevalence of post COVID-19 condition (PCC) symptoms among participants with ongoing PCC (*n* = 62) or previous PCC (*n* = 166)

The study was approved by the Swedish Ethical Review Authority (Dnr 2020–02922, Dnr 2023-03269-02). All participants provided informed consent when answering the questionnaire.

### Definition of COVID-19 and PCC

Past COVID-19 was defined based on the question “Have you ever had COVID-19?” with the response options “No”, “Yes, once”, “Yes”, several times,” and “Do not know.”

Severity of COVID-19 was defined based on the question “How sick were you during the COVID-19 infection? (in case of several infections, base your answer on the most severe)”. The response options were “no symptoms,” “mild symptoms (e.g., symptoms of cold, slight fever),” “severe symptoms (e.g., breathing difficulties, high fever, fatigue),” and “very severe symptoms (required hospital care).”

Participants who reported that they had had COVID-19 once or several times were asked about long-term symptoms through the question “Have you had long term symptoms after the COVID-19 infection (post COVID/long COVID)?” with the response options “No,” “Yes, previous symptoms but no current ongoing symptoms,” and “Yes, ongoing symptoms.” Participants who answered “Yes” to this question were asked to specify previous and ongoing long-term symptoms and their duration (< 2 months, 2–5 months, 6–12 months, or > 12 months).

The long-term symptoms included were: dyspnoea (breathing difficulties or shortness of breath); fatigue (extreme physical and/or mental tiredness); fever (or feeling feverish); altered smell or taste; headache; tachycardia (high resting heart rate or palpitations); cognitive impairment (e.g., memory and concentration difficulties); gastrointestinal problems; muscle weakness; neurological symptoms (e.g., numbness); psychiatric symptoms (e.g., depression, anxiety or feeling down); pain (e.g., chest pain or muscle and joint pain), sleep disorders, hives/urticaria, and blue-red rashes on the toes.

*PCC* was defined as at least one symptom lasting for at least 2 months after COVID-19 [9].

Participants who reported PCC were also asked about whether they had sought healthcare for their symptoms, whether they had been or currently were on sick leave for PCC, and whether they have received a PCC diagnosis.

### Definition of pre-pandemic socioeconomic,- lifestyle, and health-related factors at 24 years

Information on socioeconomic- lifestyle and health-related factors was obtained from the 24-year follow-up prior to the COVID-19 pandemic (2016–2019) (18).

*Education level* was self-reported and categorized into two categories as no university-level (including primary and lower secondary school, upper secondary school, high school, upper secondary school) or university education (including folk high school, university or college < 3 years and university or college ≥ 3 years).

*Occupation* was self-reported and categorized into three categories as student, employed, or other (including on parental leave or unpaid leave, unemployed, and other).

*Smoking* was self-reported and categorized as “Yes” (including daily or occasional smoking) or “No” (including never or previous smoking).

*Asthma* was self-reported and defined as doctor’s diagnosis of asthma (ever) together with symptoms of breathing difficulties or asthma medication occasionally or regularly in the preceding 12 months (21).

*Overweight* was determined at the clinical investigation based on measured weight and height and defined as a body mass index (BMI) ≥ 25 kg/m^2^. For most (259/268) individuals with missing information on BMI, self-reported information on weight and height was available and used to define overweight.

*Doctor diagnosed depression* was self-reported as ever having received a doctor’s diagnosis of depression.

*Wellbeing* was defined based on the question “How are you feeling right now?”. The response options “very good” and “great” were grouped and compared with the response options “good,” “quite good,” and “bad,” grouped.

*Self-perceived health* was defined based on the question “How healthy would you say you are?” The response option “completely healthy” was compared with “quite healthy” and “not very healthy”, grouped.

*Enjoying life* was defined based on the question “How happy with your life are you at the moment?”. The response options “I am very happy” and “I am mostly happy” were grouped and compared with “I am not very happy” and “I am not at all happy”, grouped.

*Physical activity* was calculated from self-reported amount (hours/week) of moderate (e.g. bicycling at normal speed, carrying light objects) and vigorous (e.g. lifting heavy weights, aerobics, or high-speed bicycling) physical activity in the last 12 months. Participants were asked about the summer and winter seasons separately and the mean of these values was calculated.

*Perceived stress* was defined using the perceived stress scale-10 (PSS-10) consisting of 10 questions on how the participant had perceived and handled stress and stressful situations in the preceding month [28]. Each question had five response options from “never” to “very often,” which were given 0 to 4 points (scores reversed for four questions including positive statements), for a total of 0–40 points.

### Definition of post-pandemic general health, stress, and changes in lifestyle factors during the pandemic

*Wellbeing*,* Self-perceived health and Enjoying life* were assessed using the same questions as in the 24-year follow-up (see above).

*Changes in lifestyle factors* during the pandemic was defined based on the question: “How do you feel that your habits/health have changed today compared with before the pandemic?” The included lifestyle factors were physical activity, sedentary time, muscle-, neck, or backpain, time spent in nature, healthy dietary habits, alcohol intake, stress, sleep, and health. The response options were “decreased a lot,” “decreased,” “unchanged,” “increased,” and “increased a lot.” For the analyses, the options “decreased a lot” and “decreased” were grouped into “decreased” and the options “increased” and “increased a lot” to “increased.”

*Perceived stress* was defined as in the 24-year follow-up.

*Physical activity* was defined as in the 24-year follow-up.

### Statistical analyses

Descriptive analyses were used to characterize the study population in terms of background factors and prevalence of previous and ongoing PCC. Differences between groups (No COVID-19 (including “No” and “Do not know”), No PCC symptoms, Previous PCC symptoms, and Ongoing PCC symptoms) were tested using the chi-squared test. Differences in median PSS-10 score and physical activity in relation to these groups were tested using the Kruskal Wallis test. Changes over time in PSS and physical activity were tested using Wilcoxon-matched-pairs signed rank test. Changes over time in general health were tested using the two-sample test of proportion. Pearson’s correlation coefficients between individual ongoing PCC symptoms and post-pandemic wellbeing were calculated. Individuals with missing data for specific variables were excluded from those analyses. All analyses were performed in Stata version 16.1 (StataCorp LLC, College Station, TX, USA). A two-sided p-value < 0.05 was considered statistically significant.

## Results

### Description of the study population and PCC symptoms

The study population (*n* = 2,098) consisted of 59.3% females with a mean age of 28.4 years (range 26.8–29.8 years).

In total, 1,577 (75.5%) reported previous COVID-19 disease (931 (44.4%) once and 646 (30.8%) several times), whereas 224 (10.7%) reported no previous disease and 297 (14.2%) reported that they did not know.

Among the participants who reported previous COVID-19 (*n* = 1,577), 166 participants (10.5%) reported previous PCC symptoms and 62 participants (3.9%) reported ongoing PCC symptoms for at least 2 months. There were some differences in sociodemographic factors between participants with PCC symptoms and participants who did not respond to the COVID-19 phase 4 questionnaire or did not report COVID-19 (Supplement Table 1).

The prevalence rate of previous and ongoing PCC were similar between females and males (10.9% vs. 10.0% and 4.1% vs. 3.7%, respectively). Those with previous and ongoing PCC more often reported having had severe or very severe COVID-19, 53.2% and 59.0%, compared with 30.4% among those with no PCC, *p* = 0.001. There was no significant difference between groups as regards pre-pandemic level of education, occupation, smoking or overweight in relation to PCC. However, the prevalence of asthma was higher among participants with previous and ongoing PCC, compared with among those without PCC or COVID-19 (Table 1).

**Table 1:** Description of pre-pandemic factors and factors assessed after the pandemic in relation to post COVID-19 condition (PCC)

	No COVID-19 (*n* = 521)	No PCC symptoms (*n* = 1,349)	Previous PCC symptoms (*n* = 166)	Ongoing PCC symptoms (*n* = 62)	*p*-value
	*n* (%)	*n* (%)	*n* (%)	*n* (%)	
**Pre-pandemic factors**					
**Sex**					0.16
- Female - Male	288 (55.3) 233 (44.7)	814 (60.3) 535 (39.7)	104 (62.7) 62 (37.4)	39 (62.9) 23 (37.1)	
**Education**					0.07
- No university-level education - University-level education	339 (65.4) 179 (34.6)	796 (59.2) 549 (40.8)	107 (64.5) 59 (35.5)	39 (62.9) 23 (37.1)	
**Occupation**					0.08
- Studying - Working - Other	275 (53.2) 190 (36.8) 52 (10.1)	781 (57.9) 483 (35.8) 85 (6.3)	89 (53.6) 67 (40.4) 10 (6.0)	31 (50.0) 25 (40.3) 6 (9.7)	
**Smoking**					0.51
- No - Yes	427 (82.3) 92 (17.7)	1093 (81.1) 254 (18.9)	141 (84.9) 25 (15.1)	48 (77.4) 14 (22.6)	
**Overweight**					0.61
- No - Yes	402 (78.1) 113 (21.9)	1030 (76.9) 310 (23.1)	122 (73.5) 44 (26.5)	46 (74.2) 16 (25.8)	
**Asthma**					0.02
- No - Yes	471 (90.6) 49 (9.4)	1195 (88.8) 151 (11.2)	137 (82.5) 29 (17.5)	51 (82.3) 11 (17.7)	
**Factors assessed after the pandemic**					
**Severe/very severe COVID-19**		410 (30.4)	98 (59.0)	33 (53.2)	< 0.001
**Sought healthcare for PCC symptoms**			21 (12.7)	19 (30.7)	0.01
**Sick leave due to PCC symptoms**					
Yes, previous sick leave			11 (6.6)	6 (9.7)	0.001
Yes, ongoing sick leave			0 (0.0)	5 (8.1)	
**PCC diagnosis**			5 (3.0)	5 (8.1)	0.10

Pre-pandemic factors were assessed in the 24-year questionnaire in 2016–2016. After the pandemic, factors were assessed in the COVID-19 phase 4 questionnaire in 2023

The most common symptom among those with ongoing PCC was altered smell and taste (*n* = 37, 59.7%), followed by psychological symptoms (*n* = 30, 48.4%) and fatigue (*n* = 27, 43.6%). Most symptoms were more prevalent among those with ongoing PCC compared with previous PCC, though this was more obvious for psychological symptoms and cognitive impairment (Fig. 1). The median number of ongoing symptoms was 3 (inter quartile range (IQR): 2–6), with a mean of 4.4 (standard deviation 3.7).

Among those with ongoing PCC, *n* = 19 (30.7%) had sought healthcare for PCC symptoms, compared with *n* = 21 (12.7%) among those with previous PCC, *p* < 0.001. Sick leave was also more common among those with ongoing PCC (*n* = 5 (8.1%) were on sick leave and *n* = 6 (9.7%) had been on sick leave, compared with 0/166 (0%) and 11/166 (6.6%), respectively, among those with previous PCC). Few participants had received a diagnosis of PCC (*n* = 5 (8.1%) of those with ongoing PCC and *n* = 5 (3.0%) of those with previous PCC) (Table 1).

### General health in relation to PCC

**Fig. 2:**
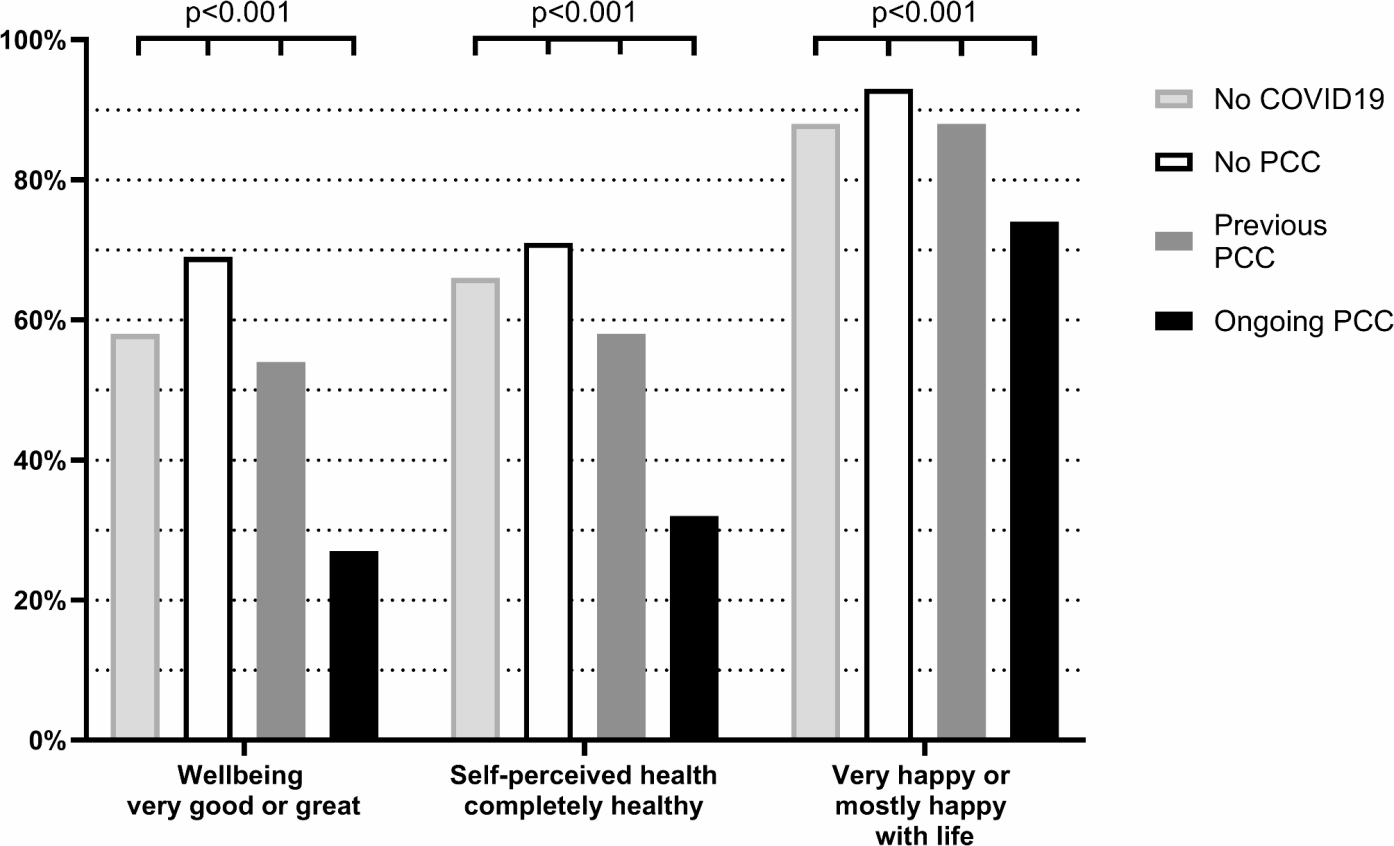
Percentage of participants reporting very good or great wellbeing, being completely healthy, and very happy or mostly happy with life, in relation to post COVID-19 condition (PCC). The p-values represent overall difference between the groups obtained by the chi-squared test

Participants with PCC, especially ongoing symptoms, reported lower post-pandemic general health compared with participants without PCC (Fig. 2). For example, 27.4% of those with ongoing PCC reported very good or great wellbeing, compared with 68.8% among those with no PCC (*p* < 0.001). This proportion was significantly lower than in the 24-year follow-up (45.2%, *p* = 0.04), whereas no significant change in longitudinal wellbeing was observed among those with no or previous PCC (Supplement Table 2). The proportions of individuals who considered themselves to be completely healthy and who were very or mostly happy with life after the pandemic were also significantly lower in the group with ongoing PCC (e.g., 32.3% vs. 70.8%, *p* < 0.001, for completely healthy) (Fig. 2). However, the differences compared with in the pre-pandemic assessment were not significant (Supplement Table 2).

Supplement Fig. 2 shows cross-sectional correlations between individual ongoing PCC symptoms and wellbeing. Fatigue was most strongly negatively correlated with wellbeing (*r*=-0.47), followed by psychiatric symptoms (*r*=-0.45), and dyspnoea (*r*=-0.36), whereas the weakest correlations were observed with hives/urticaria (*r* = 0.08) and blue-red rashes on the toes (-0.14).

The number of ongoing PCC symptoms was associated with lower self-reported health and wellbeing. Participants with ongoing PCC who reported their wellbeing as very good or great (*n* = 17) had a median of 1 symptom (IQR 1–2) compared with 4 (2–7) symptoms among those who reported lower wellbeing (*n* = 45). Among those who reported that they were completely healthy (*n* = 20), the median number of symptoms was 2, compared with 4 among those who did not report themselves as completely healthy (*n* = 42). In this group, a large majority (16/20, 80%) reported altered smell or taste as one of the symptoms.

### Changes in lifestyle factors in relation to PCC

Participants with PCC, especially ongoing PCC symptoms, were more likely to report adverse changes in lifestyle factors and health during the pandemic compared with those without PCC. Of those with ongoing PCC, 51.6% had reduced their physical activity level during the pandemic, compared with 23.4% of those without PCC and 28.7% of those with no COVID-19. Moreover, a higher proportion of this group had worsened their dietary habits (21.0% compared with 10.4% of those without PCC and 13.7% of those with no COVID-19, *p* = 0.03). Participants with ongoing PCC also reported increased muscle,- neck, or back pain (*p* = 0.02), less time spent in nature (*p* = 0.005), increased stress (*p* = 0.003), decreased sleep (*p* = 0.002), and decreased health (*p* < 0.001) (Table 2).

**Table 2:** Self-reported changes in lifestyle factors during the pandemic in relation to post COVID-19 condition (PCC). The figures are based on cross-sectional retrospective questions included in the COVID-19 phase-4 follow-up

	No COVID-19 (*n* = 521)	No PCC symptoms (*n* = 1349)	Previous PCC symptoms (*n* = 166)	Ongoing PCC symptoms (*n* = 62)	*p*-value
		*n* (%)	*n* (%)	*n* (%)	
**Physical activity**	***n*** (**%**)				
Reduced	147 (28.7)	312 (23.4)	52 (31.9)	32 (51.6)	< 0.001
Unchanged	223 (43.6)	621 (46.6)	75 (46.0)	12 (19.4)	
Increased	142 (27.7)	400 (30.0)	36 (22.1)	18 (29.0)	
**Sedentary time**					
Reduced	92 (17.9)	209 (15.7)	26 (16.0)	6 (9.7)	0.08
Unchanged	258 (50.3)	726 (54.5)	79 (48.5)	28 (45.2)	
Increased	163 (31.8)	397 (29.8)	58 (35.6)	28 (45.2)	
**Muscle**,** - neck**,** - or back pain**					
Reduced	53 (10.4)	133 (10.2)	13 (7.9)	4 (6.5)	0.02
Unchanged	312 (61.4)	815 (61.4)	86 (52.4)	31 (50.0)	
Increased	143 (28.2)	379 (28.6)	65 (39.6)	27 (43.6)	
**Time spent in nature**					
Reduced	74 (14.5)	126 (9.5)	21 (12.8)	13 (21.0)	0.005
Unchanged	275 (53.7)	711 (53.3)	90 (54.9)	30 (48.4)	
Increased	163 (31.8)	497 (37.3)	53 (32.3)	19 (30.7)	
**Healthy dietary habits**					
Reduced	70 (13.7)	134 (10.4)	25 (15.3)	13 (21.0)	0.03
Unchanged	300 (58.6)	782 (58.6)	89 (54.6)	34 (54.8)	
Increased	142 (27.7)	419 (31.4)	49 (30.1)	15 (24.2)	
**Alcohol intake**					
Reduced	159 (31.4)	453 (34.0)	67 (40.9)	27 (44.3)	0.189
Unchanged	276 (54.4)	705 (52.9)	76 (46.3)	25 (41.0)	
Increased	72 (14.2)	175 (13.1)	21 (12.8)	9 (14.8)	
**Stress**					
Reduced	65 (12.7)	195 (14.6)	14 (8.6)	3 (4.8)	0.003
Unchanged	244 (47.8)	674 (50.5)	72 (44.2)	27 (43.6)	
Increased	202 (39.5)	466 (34.9)	77 (47.2)	32 (51.6)	
**Sleep**					
Reduced	102 (20.0)	210 (15.7)	39 (23.8)	16 (25.8)	0.002
Unchanged	329 (34.4)	867 (64.9)	94 (57.3)	28 (45.2)	
Increased	80 (15.7)	258 (19.3)	31 (18.9)	18 (29.0)	
**Health**					
Reduced	114 (22.3)	236 (17.7)	49 (30.1)	37 (60.7)	< 0.001
Unchanged	284 (55.6)	766 (57.5)	78 (47.9)	13 (21.3)	
Improved	113 (22.1)	330 (24.8)	36 (22.1)	11 (18.0)	

**Fig. 3:**
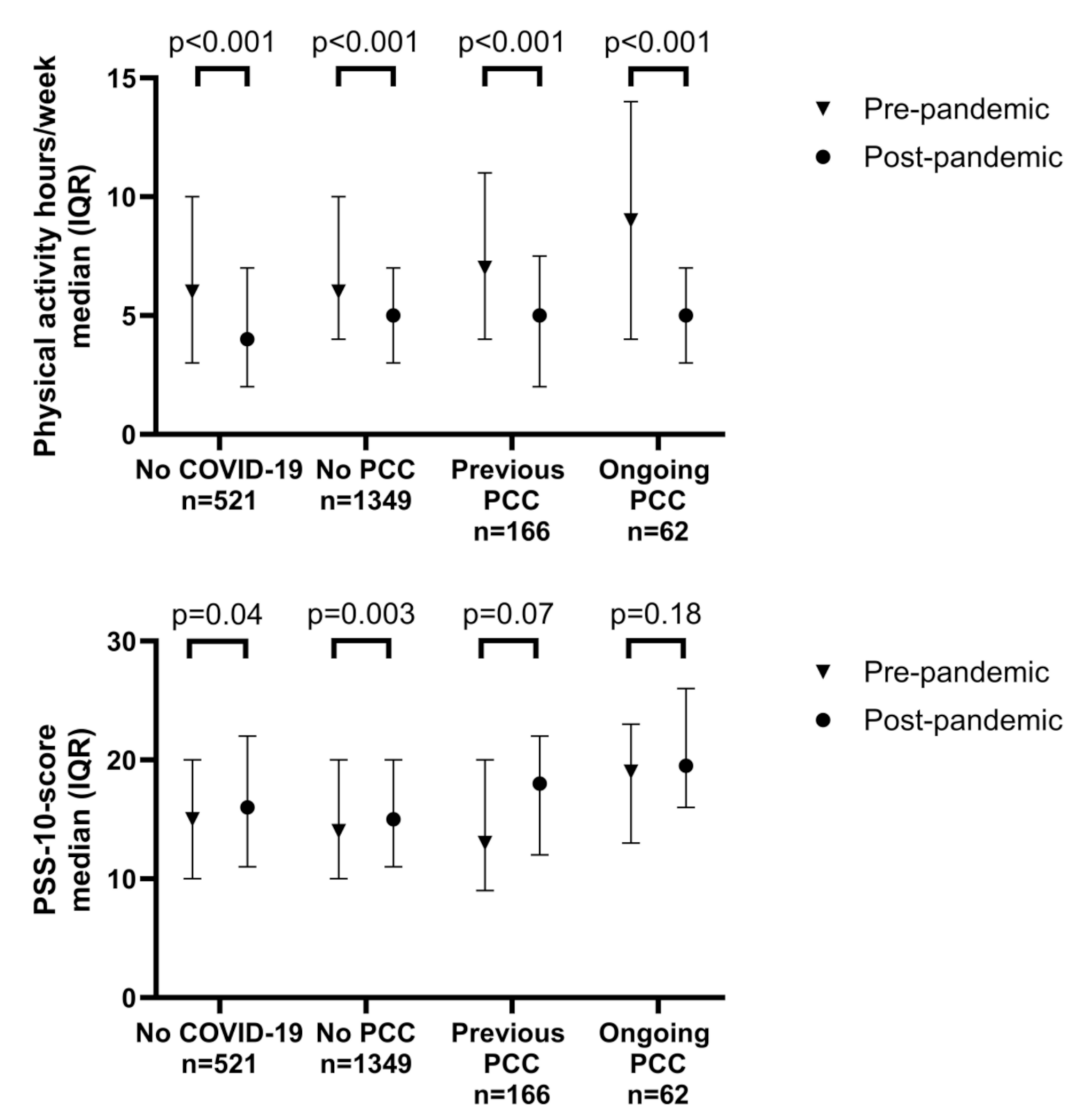
Median and inter quartile range (IQR) of perceived stress scale (PSS)-10 score (panel a) and moderate to vigorous physical activity (hours per week) (panel b) pre-pandemic (24-year-follow-up in 2016–2019) and post-pandemic (COVID-19 follow-up in 2023) in relation to COVID-19 and post COVID-19 condition (PCC). The p-values in the figures represent changes over time within the groups obtained with the Wilcoxon matched pairs-signed-rank test. Overall difference between the groups were obtained using the Kruskal-Walli’s equality-of-populations rank test (p-values not shown in the figure). The median PSS-10 score was higher both pre- (*p* = 0.02) and post-pandemic (*p* < 0.001) in the group with ongoing post COVID-19 symptoms. Median values of physical activity differed between the groups post-pandemic (*p* = 0.04), but not pre-pandemic (*p* = 0.07)

Participants with previous and ongoing PCC reported higher post-pandemic stress score (PSS-10) compared with those with no symptoms (median 18 and 19.5, compared with 15 among those with no PCC and 16 among those with no COVID-19, *p* < 0.001) (Fig. 3a). However, the group with ongoing symptoms had higher PSS-10 scores before the pandemic (median 19 vs. 14.0 among those with no PCC, *p* = 0.02) and the change over time between these groups was significant only for those with no symptoms and no COVID-19 (Fig. 3a). Regarding physical activity, all groups reported a lower amount of moderate to vigorous physical activity in the COVID-19 phase 4 follow-up questionnaire compared with before the pandemic (*p* < 0.001), with a lager reduction among those with ongoing PCC symptoms than those without PCC symptoms **(**Fig. 3b).

## Discussion

In this study, we assessed the prevalence and impact of PCC in a population-based cohort of young adults three years after the onset of the pandemic. Among those with self-reported COVID-19, the majority did not develop PCC; the prevalence of previous PCC was 10.5%, whereas 3.9% had ongoing PCC symptoms. This can be compared with a previous study within this cohort where 17% of the population with confirmed COVID-19 reported PCC at the end of 2021. To our knowledge, no study has examined the prevalence of PCC in young adults after the pandemic ended.

In the present study, we found that the group who reported ongoing symptoms had poorer wellbeing, poorer health, and more likely to report that they had undergone adverse changes in lifestyle factors. However, the group with ongoing PCC reported higher PSS, lower-self-rated health, lower well-being, and that they were less happy with life also before the pandemic. These findings suggest that establishing a causal association between PCC and lifestyle changes is challenging based on cross-sectional data alone. Nonetheless, regardless of causality, our results highlight that this group experiences significantly poorer health and well-being, emphasizing the need for targeted care and support to improve their overall health and quality of life. At the same time, almost a third of them reported themselves as “completely healthy,” despite ongoing PCC symptoms, suggesting that part of this group manages fairly well.

In contrast to in previous studies [29–31], there were no differences in socioeconomic factors, smoking, overweight or sex in relation to PCC, which may be explained by the younger population. However, as most previous studies have focused on risk factors for COVID-19, few studies have investigating how PCC itself may affect lifestyle and general health. In a review by Poudel et al. including studies of non-hospitalized patients, lower health-related quality of life after COVID-19 was associated with female sex, hospitalization, lower income, and higher age [24]. In contrast, a small cross-sectional study of young adults in Thailand did not find any differences in quality of life and body composition in relation to PCC [32].

The most common PCC symptom in our study was altered smell/taste, in line with previous findings in this population [20]. This was followed by psychological symptoms, fatigue, cognitive impairment and dyspnoea, which are among the most commonly reported symptoms in other outpatient populations [30, 33]. In contrast, a recent meta-analysis [31], where the prevalence of symptoms was assessed at different time points after the acute infection, showed that fatigue was the most common symptom reported at all timepoints assessed, from < 3 months to ≥ 12 months. Loss of taste or loss of smell were less commonly reported, at around 10% each. Hence, an unexpectedly high prevalence of altered smell/taste was observed in our study. However, according to our findings, loss of smell/taste was not strongly correlated with wellbeing.

The strengths of our study included the large population-based and well-characterized cohort with detailed information pre- and post pandemic, which enabled us to assess the impact of pre-pandemic factors and longitudinal changes in general health and some of the lifestyle factors. Further, to our knowledge, no other studies have investigated the prevalence and impact of PCC in a population-based setting of young adults over three years after the onset of the pandemic. However, the self-reported information on COVID-19, PCC symptoms and diagnosis is a limitation of our study. Moreover, there was heterogeneity in the time since the acute infection and PCC symptoms. Some of the participants might have had a recent SARS-CoV-2 infection, although the risk of prolonged symptoms seems to be reduced in later waves of COVID-19 and in vaccinated individuals [34]. In our cohort, a large majority (85.9%) of participants had been vaccinated against COVID-19 with at least one dose, and the prevalence of PCC may therefore be lower compared with in a population with lower vaccination coverage. However, in a public health perspective, the actual prevalence and symptom burden of PCC remain of interest.

## Conclusions

In conclusion, almost 4% of young adults with self-reported prior COVID-19 reported having symptoms of PCC three years after the onset of the pandemic. Participants with PCC were more likely than those without PCC to report poorer health and that lifestyle factors such as dietary habits and physical activity had deteriorated during the pandemic. However, there was no significant difference in the longitudinally measured PSS-10 score in this group. The results of the study are of public health importance as lifestyle habits can have a large impact on future health. Although the group who reported ongoing PCC is small, targeted healthcare interventions are needed to improve health in this vulnerable group.

## Electronic supplementary material

Below is the link to the electronic supplementary material.

Supplementary Material 1

Supplementary Material 2

Supplementary Material 3

Supplementary Material 4

## Data Availability

The datasets generated and/or analyzed during the current study are not publicly available due to the dataset containing sensitive personal data but are available from the corresponding author on reasonable request and with permission from Karolinska Institutet.

## Abbreviations

BMI: Body mass index
COVID-19: Corona virus disease-19
IQR: Interquartile range
PCC: Post COVID-19 condition
PSS-10: Perceived stress scale-10
SARS-CoV-2: Severe acute respiratory syndrome coronavirus 2
SD: Standard deviation
