# Supplementary material for: Post COVID-19 among young adults– prevalence and associations with general health, stress, and lifestyle factors
Source: BMC Public Health. 2025 Apr 9;25:1330. doi: 10.1186/s12889-025-22522-9 (PMC11984280; doi:10.1186/s12889-025-22522-9)
Supplement: Supplementary file 1 — Supplementary Material 1 [file 12889_2025_22522_MOESM1_ESM.docx]

| **Supplement table 2.** Pre- and post-pandemic^1^ general health in relation to post COVID-19 condition (PCC) and COVID-19 | | | | | |
| --- | --- | --- | --- | --- | --- |
|  | **No COVID-19 (n=521)** | **No PCC symptoms (n=1349)** | **Previous PCC symptoms (n=166)** | **Ongoing PCC symptoms (n=62)** |  |
| **General health variables** | **n (%)** | **n (%)** | **n (%)** | **n (%)** | **P-value^2^** |
| **Wellbeing – very good/great** |  |  |  |  |  |
| Pre-pandemic | 307 (59.6) | 875 (66.6) | 93 (57.4) | 28 (45.2) | <0.001 |
| Post-pandemic | 299 (57.6) | 925 (68.8) | 89 (53.6) | 17 (27.4) | <0.001 |
| P-value for change over time^3^ | 0.51 | 0.24 | 0.49 | 0.04 |  |
|  |  |  |  |  |  |
| **Self-perceived health – completely healthy** |  |  |  |  |  |
| Pre-pandemic | 328 (63.7) | 864 (65.9) | 90 (55.6) | 28 (45.2) | 0.001 |
| Post-pandemic | 340 (65.5) | 952 (70.8) | 97 (58.4) | 20 (32.3) | <0.001 |
| P-value for change over time^3^ | 0.54 | 0.006 | 0.60 | 0.14 |  |
|  |  |  |  |  |  |
| **Very or mostly happy with life** |  |  |  |  |  |
| Pre-pandemic | 457 (88.9) | 1229 (93.8) | 141 (87.0) | 52 (83.8) | <0.001 |
| Post-pandemic | 458 (88.3) | 1245 (92.7) | 146 (88.0) | 46 (74.2) | <0.001 |
| P-value for change over time^3^ | 0.73 | 0.29 | 0.80 | 0.19 |  |

PCC: post COVID-19 condition

^1^ Pre-pandemic factors were assessed in the 24-year questionnaire 2016-2016. Factors assessed after the pandemic were assessed in the COVID-19 phase 4 questionnaire in 2023.

^2^ P-value obtained by chi-2 test

^3^ P-value obtained by Two-sample test of proportions
