# Supplementary material for: Post COVID-19 among young adults– prevalence and associations with general health, stress, and lifestyle factors
Source: BMC Public Health. 2025 Apr 9;25:1330. doi: 10.1186/s12889-025-22522-9 (PMC11984280; doi:10.1186/s12889-025-22522-9)
Supplement: Supplementary file 2 — Supplementary Material 2 [file 12889_2025_22522_MOESM2_ESM.docx]

| **Table Supplement table 1.** Description of pre-pandemic^1^ factors in different groups of participants and in non-participants | | | | | | |
| --- | --- | --- | --- | --- | --- | --- |
|  | **Did not respond to the COVID-19 phase 4 follow-up (n=1,988)** | **Reported no prior infection at the COVID-19 phase 4 follow-up (n=521)** | **No PCC symptoms at the COVID-19 phase 4 follow-up (n=1,349)** | **Previous PCC symptoms (n=166)** | **Ongoing PCC-symptoms at phase 4 (n = 62)** |  |
| **Categorical variables** | **n (%)** | **n (%)** | **n (%)** | **n (%)** | **n (%)** | **P-value^2^** |
| **Sex**   - Female - Male | 778 (39.1)  1210 (60.9) | 288 (55.3)  233 (44.7) | 814 (60.3)  535 (39.7) | 104 (62.7)  62 (37.4) | 39 (62.9)  23 (37.1) | <0.001 |
| **Education**   - No university education - University education | 669 (70.3)  283 (29.7) | 339 (65.4)  179 (34.6) | 796 (59.2)  549 (40.8) | 107 (64.5)  59 (35.5) | 39 (62.9)  23 (37.1) | <0.001 |
| **Occupation**   - Studying - Working - Other | 386 (40.3)  484 (50.6)  87 (9.1) | 275 (53.2)  190 (36.8)  52 (10.1) | 781 (57.9)  483 (35.8)  85 (6.3) | 89 (53.6)  67 (40.4)  10 (6.0) | 31 (50.0)  25 (40.3)  6 (9.7) | <0.001 |
| **Smoking**   - No - Yes | 711 (74.1)  248 (25.9) | 427 (82.3)  92 (17.7) | 1093 (81.1)  254 (18.9) | 141 (89.9)  25 (15.1) | 48 (77.4)  14 (22.6) | <0.001 |
| **Overweight**   - No - Yes | 696 (73.1)  256 (26.9) | 402 (78.1)  113 (21.9) | 1030 (76.9)  310 (23.1) | 122 (73.5)  44 (26.5) | 46 (74.2)  16 (25.8) | 0.16 |
| **Asthma**   - No - Yes | 858 (89.1)  105 (10.9) | 471 (90.6)  49 (9.4) | 1195 (88.8)  151 (11.2) | 137 (82.5)  29 (17.5) | 51 (82.3)  11 (17.7) | 0.03 |
| **Self-perceived health**   - Not completely healthy - Completely healthy | 351 (41.1)  503 (58.9) | 187 (36.3)  328 (63.7) | 448 (34.2)  864 (65.9) | 72 (44.4)  90 (55.6) | 34 (54.8)  28 (45.2) | <0.001 |
| **Continuous variables** | **Median (IQR)** | **Median (IQR)** | **Median (IQR)** | **Median (IQR)** | **Median (IQR)** | **P-value^3^** |
| **Physical activity, moderate to vigorous (h/week)** | 7 (4-14) | 6 (3-10) | 6 (4-10) | 7 (4-11) | 9 (4-14) | <0.001 |
| **PSS-10 score** | 16 (11-21) | 15 (10-20) | 14 (10-20) | 13 (9-20) | 19 (13-23) | 0.02 |

Pre-pandemic factors were assessed in the 24-year questionnaire 2016-2016.

IQR: inter quartile range

PCC: post COVID-19 syndrome

PSS: perceived stress scale

2 P-value obtained by chi-2 test

3 P-value obtained by Kruskal-Wallis test
