# Supplementary figures and images for: Post COVID-19 among young adults– prevalence and associations with general health, stress, and lifestyle factors
Source: BMC Public Health. 2025 Apr 9;25:1330. doi: 10.1186/s12889-025-22522-9 (PMC11984280; doi:10.1186/s12889-025-22522-9)

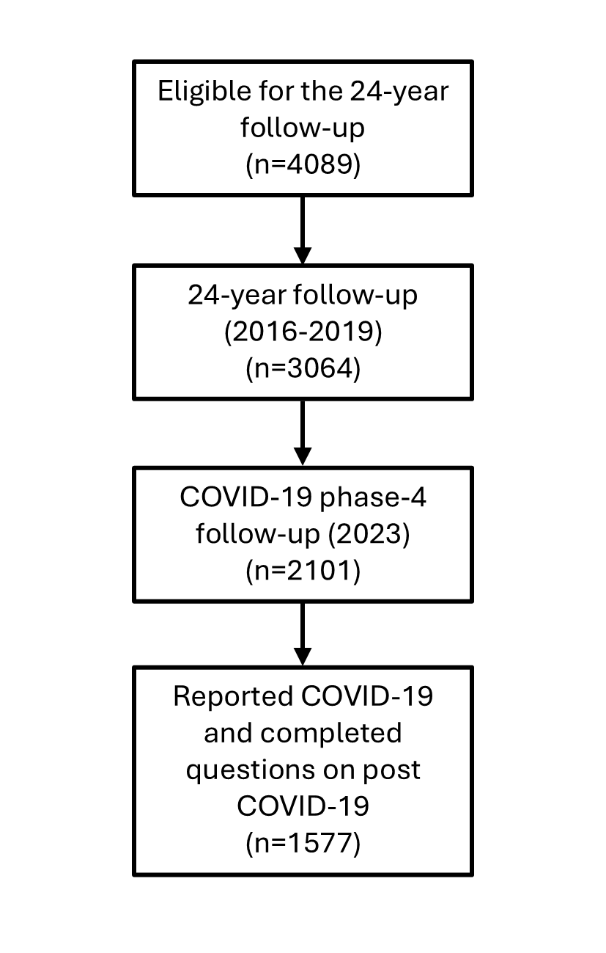


**Supplement Figure 1.** Flowchart of the study.

Supplement: Supplementary file 3 — Supplementary Material 3 [file 12889_2025_22522_MOESM3_ESM.docx]
