# Supplementary material for: Post COVID-19 among young adults– prevalence and associations with general health, stress, and lifestyle factors
Source: BMC Public Health. 2025 Apr 9;25:1330. doi: 10.1186/s12889-025-22522-9 (PMC11984280; doi:10.1186/s12889-025-22522-9)
Supplement: Supplementary file 4 — Supplementary Material 4 [file 12889_2025_22522_MOESM4_ESM.docx]

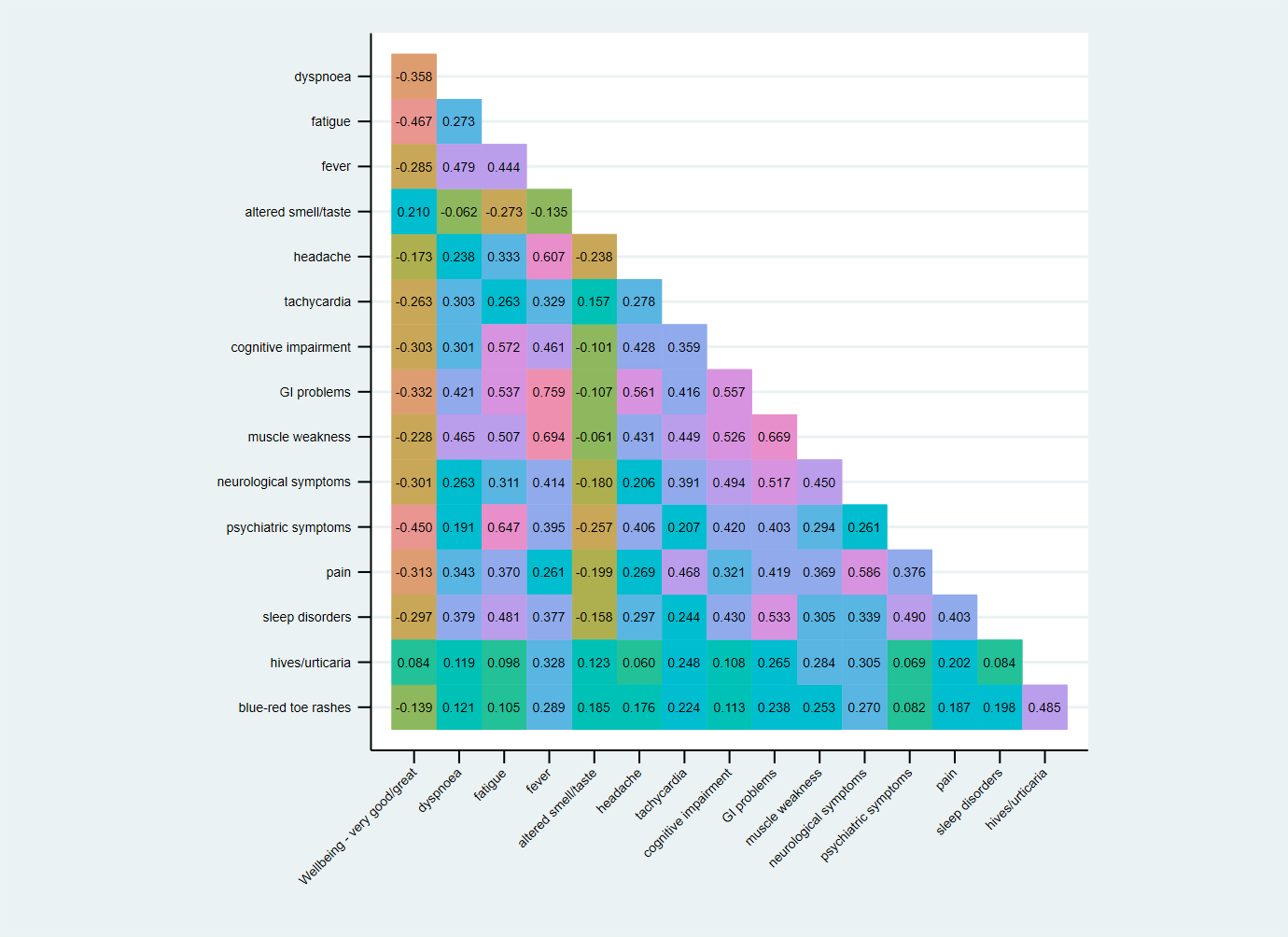


**Supplement Figure 2.** Correlation plot. The plot shows Pearson’s correlation coefficients between ongoing post COVID-19 symptoms and wellbeing categorized as “very good”/ “great” compared with “good”/“quite good”/“bad.” GI: gastrointestinal.
